# Gasless endoscopic thyroidectomy via the trans-subclavian approach versus conventional open thyroid surgery for unilateral thyroid cancer

**DOI:** 10.1038/s41598-026-42491-2

**Published:** 2026-03-06

**Authors:** Rui Yang, Peng Han, Chi Ma, Yanzhong Gu, Hongji Wu, Haiqing Sun, Shujian Wei, Xincheng Liu, Haitao Zheng

**Affiliations:** 1 Shapingba Hospital affiliated to Chongqing University(Shapingba District People’s Hospital of Chongqing), No. 2, Jialang Road, Shapingba District, Chongqing, China 400030; 2 Yantai Yuhuangding Hospital, Yantai, 264000 Shandong China; 3 School of Clinical Medicine, Shandong Second Medical University, Weifang, Shandong China; 4 The Second School of Clinical Medicine of Binzhou Medical University, Yantai, Shandong China

**Keywords:** Papillary thyroid carcinoma, Thyroid surgery, Endoscopy, Trans-subclavian approach, Conventional open thyroid surgery, Cancer, Thyroid cancer, Endocrine system and metabolic diseases, Thyroid cancer, Thyroid cancer, Thyroid diseases

## Abstract

The incidence of papillary thyroid carcinoma (PTC) is increasing worldwide. With advancements in society and technology, the variety of thyroid endoscopic surgeries is expanding. Gasless endoscopic thyroidectomy via the trans-subclavian approach (GETTSA) has been used for many years; yet, relevant research data are limited. This study aimed to assess the reliability of GETTSA by comparing it with conventional open thyroid surgery (COT). Clinical data were collected from 488 patients who underwent unilateral PTC surgery at Yantai Yuhuangding Hospital. Based on propensity score matching, relevant data were compared between the two procedures using the chi-square test, independent-sample t-test, and Mann-Whitney U test. COT and GETTSA significantly differed with respect to Thyroid Cancer-Specific Quality of Life (*P* < 0.001), scar appearance (*P* = 0.031), swallowing function (*P* = 0.001), operative time (*P* < 0.001), hospital fees (*P* < 0.001), number of central lymph node dissections (*P* < 0.001), drainage volume on the first postoperative day (*P* = 0.017), and parathyroid autotransplantation(*P* = 0.019). However, no notable differences in the number of lymph node metastases (*P* = 0.155), length of postoperative hospital stay (*P* = 0.181), or transient vocal cord paralysis (*P* = 0.478) were observed between COT and GETTSA. Additionally, no complications (secondary surgery, tracheal fistula, poor healing, or postoperative bleeding) occurred in either group. In conclusion, GETTSA is a reliable surgical procedure with the help of carbon nanoparticles; however, attention should be paid to protecting the parathyroid glands.

## Introduction

The incidence of thyroid cancer has rapidly increased recently^1^. Surgery remains the most effective treatment for thyroid cancer, with various types available^2^. Despite conventional open thyroid surgery (COT) being considered the “gold standard,” it has drawbacks, such as noticeable scarring and swallowing discomfort, which will impact the quality of life of thyroid cancer patients^3,4^. Evolving patient expectations and advancements in living standards have increased the demand for surgical options beyond COT, leading to the development of various endoscopic thyroidectomy techniques. These approaches each have distinct advantages and limitations. For example, transoral endoscopic surgery offers excellent cosmetic results; however, it is limited by oral cavity anatomy, resulting in a relatively narrow operative space, which may increase intraoperative risks. Although axillary endoscopic surgery can achieve a scar-free face, the approach is more distant, requiring extensive tissue dissection, which often leads to longer operation times. Gasless endoscopic thyroidectomy via the trans-subclavian approach (GETTSA), introduced by Shimizu in 1998, offers advantages such as an ample operative space, clear visualization, and cosmetically favorable scars. Although it has been adopted in several hospitals^5–8^, there remains a lack of robust evidence regarding its safety, oncologic thoroughness, and impact on patient quality of life. Hence, we aimed to conduct a comparative study of GETTSA and COT for unilateral papillary thyroid carcinoma (PTC) treatment based on propensity score matching (PSM), so as to demonstrate that GETTSA can safely and effectively resect the tumor and is beneficial to the quality of life of patients.

## Methods

### Selection of study participants

This study was conducted in the Department of Thyroid Surgery at Yantai Yuhuangding Hospital and was approved by the Ethics Committee of Yantai Yuhuangding Hospital. All patients signed a surgical consent form and informed consent for participation in our research. This study was conducted in accordance with Nature Portfolio journals’ policies, and all methods were performed in accordance with the relevant guidelines and regulations. All patients underwent surgery between December 2022 and August 2023, and the same team performed all procedures. The patients were exhaustively informed about the advantages and disadvantages of all available thyroidectomy approaches before surgery, and they were allowed to carefully and freely select their preferred approach. The inclusion criteria were as follows: (1) diagnosis of PTC with tumor maximal diameters ≤ 4 cm and (2) surgery for unilateral thyroid carcinoma( including the affected lobe of the thyroid gland, the isthmus, and the central neck lymph nodes.). The exclusion criteria were as follows: (1) lateral cervical lymph node metastasis, (2) tumors invading adjacent organs, (3) history of neck radiation therapy, (4) absence of statistical data, (5) ectopic thyroid, (6) history of neck surgery, and (7) patients undergoing day surgery. In GETTSA, we used an ultrasonic scalpel(Johnson & Johnson) and Hemlock clips(KANGJI), whereas in COT, conventional electrocautery and suture ligation were primarily used for vessel sealing.

### Sample size calculation

We utilized Zstats to perform the sample size calculation. Data from 20 patients who underwent GETTSA and 20 patients who underwent COT were used, with the central compartment lymph node metastasis rate serving as the incidence rate for the exposed (GETTSA) and control (COT) groups (0.25 and 0.20, respectively). Setting a significance level (α) of 0.05 and a power (1-β) of 0.8, the calculation indicated that a minimum of 24 patients per group (GETTSA and COT) would be necessary to achieve statistical validity.

### Observation indicators

This retrospective study included 488 patients who underwent unilateral PTC surgery at the Department of Thyroid Surgery of Yantai Yuhuangding Hospital between December 2022 and August 2023. Medical records were retrospectively reviewed to collect data related to patient age, sex, underlying disease, body mass index (BMI), chronic lymphocytic thyroiditis, duration of surgery, drainage volume on the first postoperative day, number of central lymph node dissections, number of central lymph node metastases, hospitalization costs, length of hospitalization, transient vocal cord paralysis paralysis, incision dehiscence, tracheal fistula, postoperative bleeding, and parathyroid autotransplantation. We used both visual assessment and autofluorescence imaging of the excised specimens to determine whether inadvertent resection of the parathyroid glands occurred. In cases of inadvertent removal, we actively performed parathyroid autotransplantation. For GETTSA, carbon nanoparticle suspension was injected preoperatively under ultrasound guidance, whereas for COT, the injection was performed intraoperatively. Laryngoscopy was performed postoperatively only in patients who reported symptoms such as hoarseness or coughing, and not routinely in asymptomatic patients. To investigate the patients’ postoperative quality of life, we used the Thyroid Cancer-Specific Quality of Life^9^ (THYCA-QOL) scale between February and March postoperatively. The content of this scale includes items assessing symptoms such as dry mouth, difficulty swallowing, hoarseness, weakness in speech, foreign body sensation in the throat, annoyance with neck scars, sensitivity to cold and heat, etc. Each item is scored 1, 2, 3, 4 or 5 according to the patient’s condition. Specific items regarding neck scar discomfort were used to evaluate surgical scarring, while items on dysphagia and throat lump sensation were used to assess postoperative swallowing function. Additionally, the total THYCA-QOL score was calculated.

### Statistical analysis

**Fig. 1:**
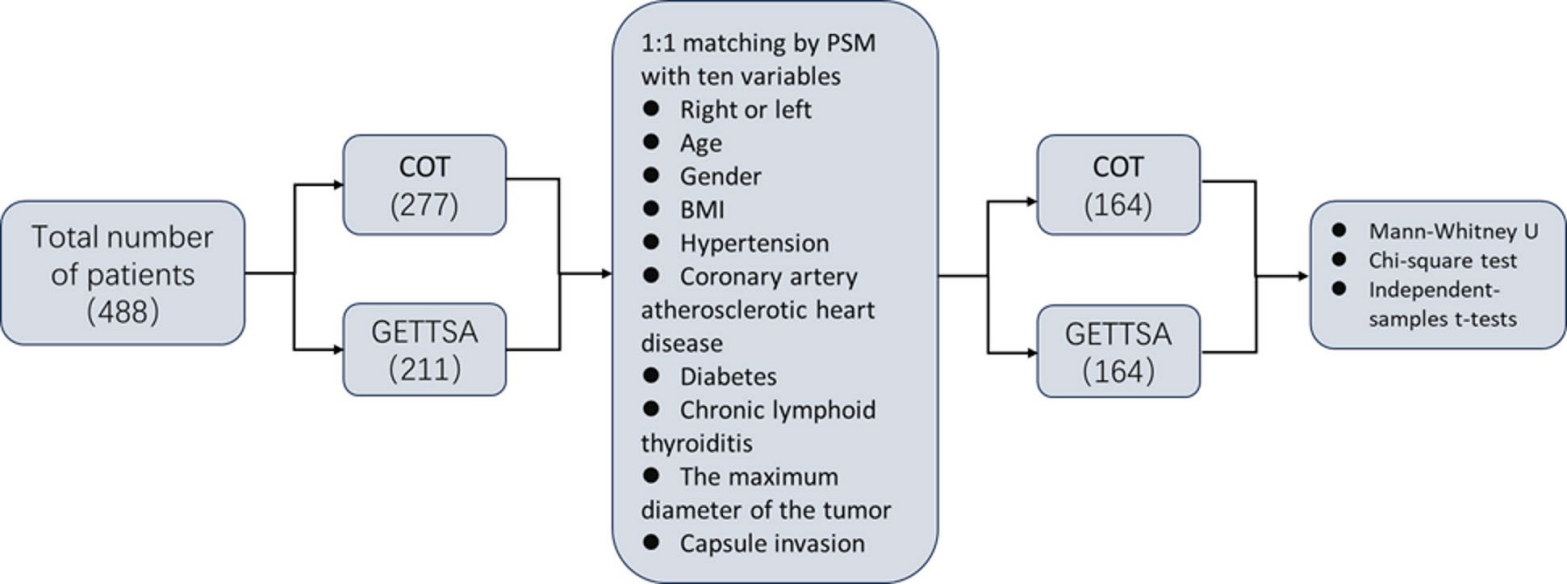
Process of analysis.

PSM was performed using SPSS software version 26.0 to minimize patient selection bias and adjust for differences in baseline clinicopathological characteristics. The following ten clinical information items of patients that might affect surgical outcomes were selected as covariates: patient age, BMI, sex, tumor site, maximum tumor diameter, hypertension, diabetes, coronary heart disease, chronic lymphocytic thyroiditis, and whether the capsule was violated. After including all the aforementioned variables, 1:1 PSM was performed using the nearest-neighbor method with a caliper width of 0.05, which is the standard deviation of the logit of the propensity score, resulting in 164 patient pairs that were successfully matched. After matching, between-group comparisons were performed using SPSS. Continuous normally distributed variables were expressed as mean ± standard deviation and compared with the independent-sample t-test. Categorical variables were analyzed with the chi-square test. Non-normally distributed continuous variables were expressed as median (interquartile range) and compared with the Mann-Whitney U test Statistical significance was defined as *P* < 0.05 (Fig. 1).

### Process of surgery

**Fig. 2:**
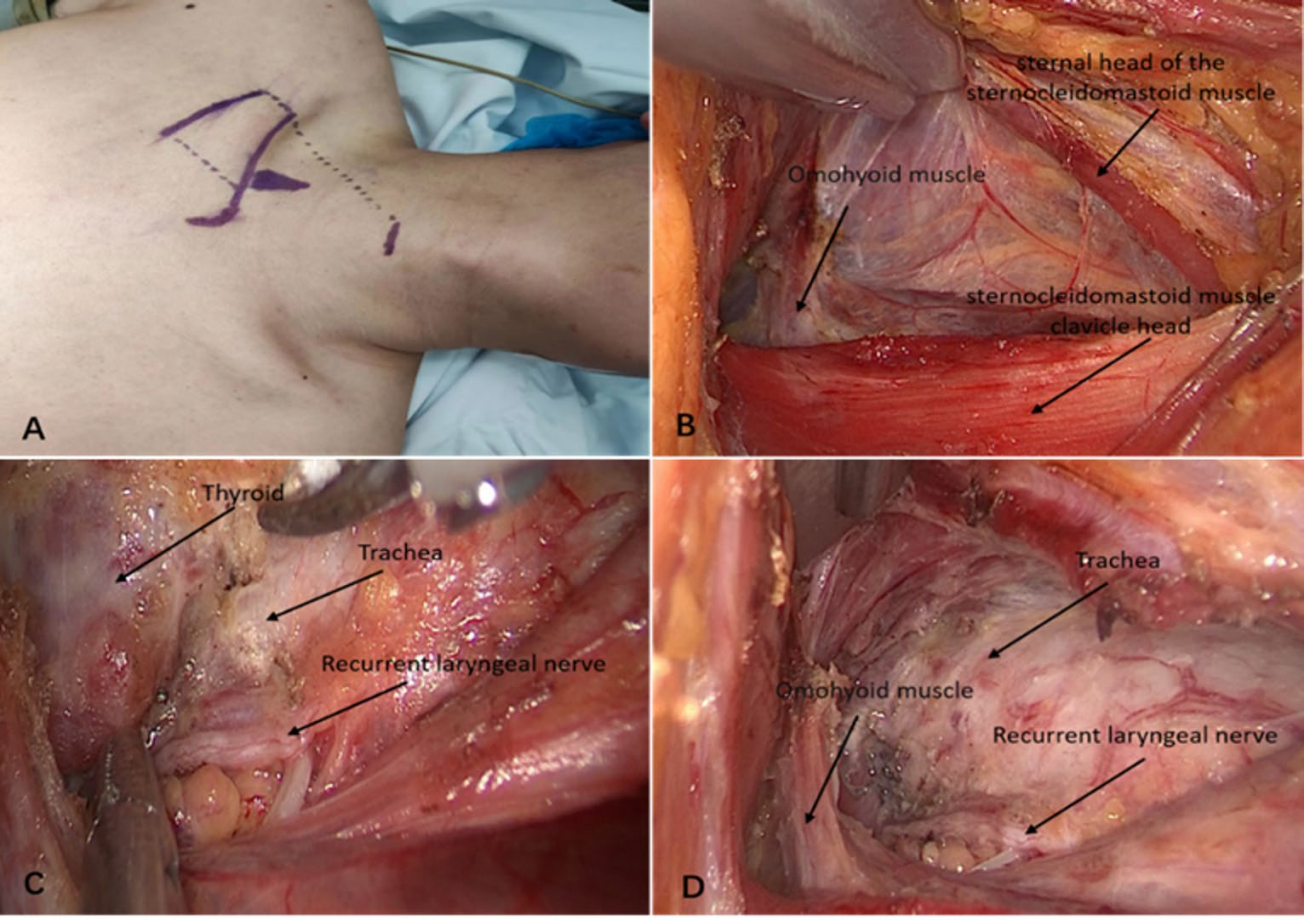
(**A**) Position and marking for surgery. (**B**) After lifting the strap muscles. (**C**) After freeing the recurrent laryngeal nerve. (**D**) After complete resection of the lesion.

The COT surgical procedure has been previously reported and is well known. The GETTSA procedure was performed as follows: the patients were placed in the supine position under general anesthesia. The patient’s head faced the opposite side of the procedure (Fig. 2A), and the surgical area was disinfected. An approximately 5-cm incision was made along the skin lines below the midpoint of the clavicle, and a flap was created. By separating the sternal head of the sternocleidomastoid muscle from the clavicular head and inserting a special suspension hook, the sternal head of the sternocleidomastoid muscle was lifted. Fat and lymphoid tissues were cleaned using an ultrasonic knife. The superficial layer of the deep carotid fascia between the strap muscle and carotid sheath was opened using an ultrasonic knife after exposing and freeing the scapulohyoid muscle. After adjusting the suspension hook, the strap muscle was lifted, exposing the thyroid area (Fig. 2B). The recurrent laryngeal nerve and parathyroid gland were explored and protected by dissection along the medial border of the carotid artery-prevertebral fascia–esophagus level using an ultrasonic knife (Fig. 2C). After disconnecting the inferior thyroid artery, the thyroid gland was pulled to the opposite side to expose its dorsal side of the thyroid glands and detect the entry of the recurrent laryngeal nerve into the larynx. The parathyroid gland and fat pad were separated from the thyroid gland. The upper pole of the thyroid gland was exposed by pulling, and the vessels were clampedand severed. The thyroid gland was pulled inward, covering the recurrent laryngeal nerve with gauze and separating it from the trachea. Subsequently, the thyroid isthmus was cut, and the specimens were carefully removed and examined for the parathyroid glands. The central lymph node was removed after intraoperative pathological confirmation of PTC. Finally, the wound was flushed (Fig. 2D), and the incision was sutured after confirming the absence of active bleeding.

## Results

### Baseline information

Overall, 488 eligible patients underwent surgery, with 277 selecting COT and 211 opting for GETTSA. Following 1:1 propensity score matching (PSM), 164 well-balanced patient pairs were generated. No significant differences were observed between the two groups in terms of sex (*P* = 0.532), tumor site (*P* = 0.825), hypertension (*P* = 0.822), coronary heart disease (*P* > 0.999), diabetes (*P* = 0.358), chronic lymphocytic thyroiditis (*P* = 0.903), capsule invasion (*P* = 0.810), age [COT, 43.00 (36.00–51.00) vs. GETTSA, 42.00 (35.00–51.75), Z=-0.707, *P* = 0.479], BMI [COT, 25.40 (23.03–28.18) vs. GETTSA, 24.80 (22.60–28.70), Z=-0.816, *P* = 0.415], and maximum tumor diameter [COT, 0.75 (0.50–1.00) vs. GETTSA, 0.70 (0.50–1.00), Z=-0.355, *P* = 0.722] (Table 1).

**Table 1:** Basic information.

Variable	COT	GETTSA	Z/X^2^	*P*	SMD
Age (years)	51.00(40.00–58.00)	40.00(33.00–48.00)	-8.317	<0.001	-0.893
Body mass index (kg/m^2^)	25.50 (23.40–28.30)	24.70 (22.50–28.40)	-2.033	0.042	-0.145
Maximum tumor diameter (cm)	0.800(0.500–1.100)	0.70 (0.50–1.00)	-2.128	0.033	-0.171
Tumor location			0.201	0.654	-0.041
Right	144	114			
Left	133	97			
Sex					-0.015
Male	70	52	0.025	0.874	
Female	207	159			
Hypertension			22.761	0.000	-0.675
Yes	56	11			
No	221	200			
Coronary artery atherosclerotic heart disease			6.107	0.013	-0.509
Yes	11	1			
No	266	210			
Diabetes			7.261	0.007	-0.390
Yes	257	207			
No	20	4			
Chronic lymphocytic thyroiditis			0.000	0.999	0.000
Yes	84	147			
No	193	64			
Thyroid capsule invasion			5.036	0.025	-0.197
Yes	201	133			
No	76	78			

### THYCA-QOL, hospitalization, and surgical information

Noticeable differences were observed in the total score [COT, 42.00 (39.00–44.00) vs. GETTSA, 39.00 (37.25–42.00), Z=-5.837, *P* < 0.001], swallowing function [COT, 3.00 (3.00–4.00) vs. GETTSA, 3.00 (3.00–3.00), Z=-2.161, *P* = 0.031], and scar evaluation [COT, 1.00 (1.00–2.00) vs. GETTSA, 1.00 (1.00–1.00), Z=-3.322, *P* = 0.001] (Table 2). Furthermore, the results showed significant differences in patient operative time [COT, 80.00 (65.00–93.75) vs. GETTSA, 110.00 (90.00–130.00), Z=-10.364, *P* < 0.001], drainage volume on the first postoperative day [COT, 45.00 (35.00–50.00) vs. GETTSA, 49.00 (40.00–60.00), Z=-2.388, *P* = 0.017], and hospitalization costs (21155.45 ± 2887.73 vs. 23340.14 ± 1900.95, *P* < 0.001). However, no significant differences in postoperative hospital stay [COT, 1.00 (1.00–3.00) vs. GETTSA, 1.00 (1.00–2.00), Z=-1.339, *P* = 0.181] were noted (Table 3).

**Table 2:** Basic information following propensity score matching.

Variable	COT	GETTSA	Z/X^2^	*P*	SMD
Age (years)	43.00 (36.00–51.00)	42.00 (35.00–51.75)	-0.707	0.479	-0.112
Body mass index (kg/m^2^)	25.40 (23.03–28.18)	24.80 (22.60–28.70)	-0.816	0.415	-0.055
Maximum tumor diameter (cm)	0.75 (0.50–1.00)	0.70 (0.50–1.00)	-0.355	0.722	-0.058
Tumor location			0.049	0.825	0.024
Right	89	87			
Left	75	77			
Sex			0.391	0.532	-0.070
Male	46	41			
Female	118	123			
Hypertension			0.051	0.822	0.024
Yes	154	153			
No	10	11			
Coronary artery atherosclerotic heart disease			-	> 0.999	0.078
Yes	0	1			
No	164	163			
Diabetes			0.847	0.358	-0.119
Yes	7	4			
No	157	160			
Chronic lymphocytic thyroiditis			0.015	0.903	-0.013
Yes	48	47			
No	116	117			
Thyroid capsule invasion			0.058	0.810	0.027
Yes	113	115			
No	51	49			

**Table 3:** THYCA-QOL total, swallowing function, and scarring scores.

Variable	COT	GETTSA	Z	*P*	SMD
Total score	42.00 (39.00–44.00)	39.00 (37.25–42.00)	-5.837	< 0.001	-0.271
Swallowing score	3.00 (3.00–4.00)	3.00 (3.00–3.00)	-2.161	0.031	-0.303
Scar score	1.00 (1.00–2.00)	1.00 (1.00–1.00)	-3.322	0.001	

THYCA-QOL, Thyroid Cancer-Specific Quality of Life.

### Surgical completeness

Surgical completeness was assessed based on the number of central lymph nodes retrieved and the number of central lymph node metastases. The two groups showed a significant difference in the number of central lymph node dissections [COT, 8.00 (6.00–11.75) vs. GETTSA, 4.00 (2.00–7.75); Z=-7.003; *P* < 0.001]. However, there was no significant difference in the number of central lymph node metastases [0.00 (0.00–1.00) vs. 0.00 (0.00–1.00), Z=-1.422, *P* = 0.155] (Table 3).

### Postoperative complication

The chi-square test revealed no significant difference in transient vocal cord paralysis (*P* = 0.478). However, there was a significant difference in parathyroid autotransplantation(*P* = 0.019). Additionally, no patient exhibited poor incision healing, postoperative bleeding, tracheal fistula, or the need for secondary surgery (Table 4). In this study, all remaining patients who did not undergo PSM subsequently underwent successful and uncomplicated surgery, with good postoperative recovery.

**Table 4:** Hospitalization and surgical characteristics.

Variable	COT	GETTSA	Z/t	*P*
Operative time	80.00 (65.00–93.75)	110.00 (90.00–130.00)	-10.364	< 0.001
Postoperative length of stay	1.00 (1.00–3.00)	1.00 (1.00–2.00)	-1.339	0.181
Hospital fees	21155.45 ± 2887.73	23340.14 ± 1900.95	-8.092	< 0.001
Lymph node dissection	8.00 (6.00–11.75)	4.00 (2.00–7.75)	-7.003	< 0.001
Lymph node metastases	0.00 (0.00–1.00)	0.00 (0.00–1.00)	-1.422	0.155
1st flow diversion	45.00 (35.00–50.00)	49.00 (40.00–60.00)	-2.388	0.017

## Discussion

Currently, endoscopic thyroid surgery encompasses several methods, including transoral, transaxillary, anterior chest, subclavian, and postauricular approaches^7,10–13^. The transoral approach has attracted attention due to its natural advantage of hidden scars, but it poses certain challenges in terms of technical difficulty and potential risk of infection. The transaxillary approach, on the other hand, offers a larger operative space and better visualization, enhancing safety, but it involves greater trauma and longer operative time. GETTSA shows promising prospects due to its high patient acceptance, easier operation, and smaller surgical trauma. Our study was conducted using PSM to rigorously evaluate GETTSA.

### Quality of life and surgical scarring

**Fig. 3:**
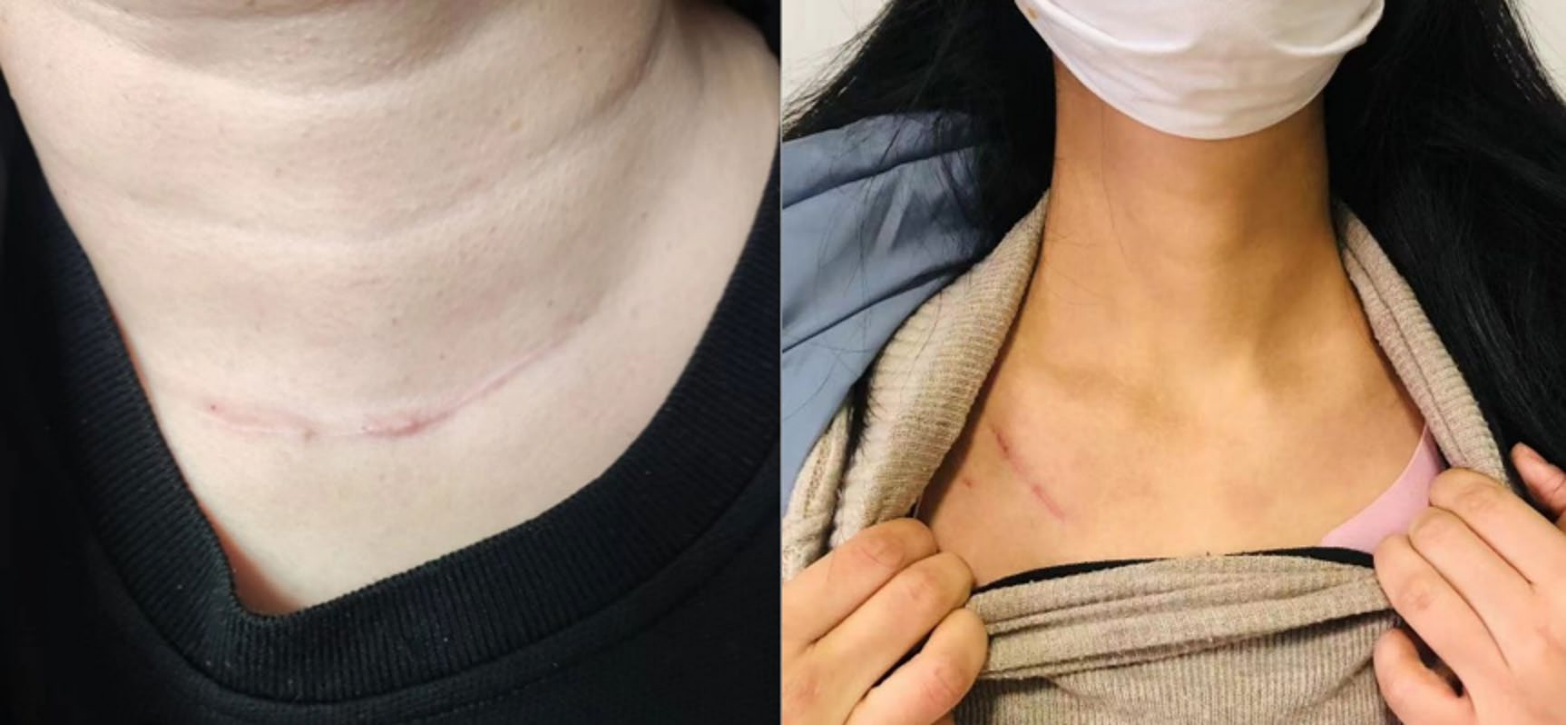
Postoperative scarring. Left: Scarring after COT. Right: Scarring after GETTSA.

Studies have shown that COT can affect the postoperative quality of life, and some scholars have demonstrated a negative correlation between the patient scar assessment scale and the quality-of-life score^14–17^. GETTSA improved postoperative quality of life, with a minor impact on patients’ daily living compared to COT, primarily owing to better swallowing and a relatively concealed incision site, reducing the impact on daily life and psychological pressure on patients (Fig. 3). Li et al. reported a close relationship between the swallowing function and strap muscles^18^. The GETTSA does not damage the strap muscles because the surgical cavity is built through the natural anatomical space. However, a comparative study found no significant difference in swallowing function between patients undergoing endoscopic (*n* = 31) and COT (*n* = 60), even though the endoscopic procedure avoided strap muscle dissection^19^.

### Hospitalization information

This and previous studies did not find significant differences in the postoperative stay length between GETTSA and COT^8^. However, our study indicated higher hospitalization costs for thyroid endoscopic surgery than for COT, consistent with the findings of Zhu et al.^8^. This cost disparity may be attributed to the increased use of consumables and equipment during endoscopic surgery. GETTSA is associated with a longer operative duration than COT. This is a common phenomenon in endoscopic thyroid surgery, including a modified gasless trans-subclavian approach^8,10,20^. The underlying reasons for this phenomenon are as follows: (1) Establishing a surgical space in GETTSA requires an incision from the lower edge of the clavicle, potentially leading to increased time. (2) The instruments used in thyroid endoscopic surgery are more intricate for surgeons than those used in COT. (3) Moreover, the use of an endoscope can pose challenges in confined workspaces. Nevertheless, the surgery time may decrease as the operator’s proficiency increases.

An increase in free tissue often correlates with increased postoperative drainage. Numerous comparative studies have noted that various endoscopic thyroidectomy approaches (e.g., areola, transoral) create more free tissue and consequently result in higher postoperative drainage volumes than COT^20–22^. GETTSA, which also involves creating more free tissue, typically results in greater drainage on the initial postoperative day than COT. Previous research has also indicated that patients with stage cN0 unilateral thyroid carcinoma undergoing subclavian treatment exhibit higher postoperative drainage levels than those undergoing COT^8^.

### Surgical thoroughness

Although the 2015 ATA guidelines^23^ remain cautious regarding patients with PTC smaller than 1 cm, the decision should be considered in conjunction with the probability of lymph node metastasis in individual patients. Notably, node metastasis is very common in PTC patients^24,25^. In patients who underwent central cervical lymph node dissection, the metastasis rate was 82.3%. Residual metastatic lymph nodes may increase the risk of recurrence^23,26^. Therefore, effective dissection of the central lymph nodes during thyroid surgery can reduce postoperative recurrence and improve prognosis. Compared to open surgery, the phenomenon of fewer central lymph nodes being cleared by endoscopic thyroid surgery is common^20,27^. Our study revealed a difference in the number of central lymph nodes dissected by COT and GETTSA. This may reflect selection bias in our cohort, in which patients chosen for GETTSA tended to have clinical features associated with a lower extent of central neck disease, and the clavicular obstruction encountered with GETTSA further increased the difficulty of dissection. Additionally, during COT, there may be extra dissection of lymph nodes around the left recurrent laryngeal nerve in front of the trachea. Moreover, much of the lymphatic fat in the contralateral central neck may be removed as the pretracheal region retracts during COT, potentially increasing the number of lymph nodes dissected, emphasizing the importance of careful evaluation of patients’ lymph node conditions before surgery. We contend that relying solely on lymph node metastasis as an indication for GETTSA is not appropriate. The surgeon must comprehensively evaluate the specific status of nodal disease. We propose that GETTSA should be reserved for patients in whom preoperative imaging reveals no evidence of lymph node metastasis, or in whom metastatic lymph nodes are confined to a region that is predicted to be amenable to complete dissection via the endoscopic approach. Conversely, GETTSA is contraindicated in cases with extensive or unpredictable cervical lymph node metastasis, imaging findings suggesting potentially inaccessible lesions, or anatomical constraints that would prevent thorough endoscopic dissection. This includes, but is not limited to, the presence of metastasis in lymph nodes located below the sternal level. Notably, no significant difference was observed in the number of dissected central lymph node metastases (*P* = 0.155). Wang et al. reported that the application of carbon nanoparticles improves the thoroughness of central neck dissection in endoscopic thyroid surgery^28^. In our department, carbon nanoparticles are used for GETTSA and COT, and this practice indicates that GETTSA can effectively identify metastatic lymph nodes with the help of tracers, thereby enhancing surgical thoroughness.

### Postoperative complications

Postoperative complications, such as postoperative hemorrhage, tracheal fistula, transient vocal cord paralysis, and parathyroid gland issues, are critical for evaluating surgical safety. Our study identified two cases (1.2%) of transient vocal cord paralysis using GETTSA. This rate is comparable to that of many previously reported types of thyroid procedures (Table 5), such as the anterior chest approach (1.3%), gasless endoscopic transaxillary thyroidectomy (0.9%), endoscopic thyroidectomy via the areola approach (2.6%), transoral endoscopic thyroidectomy vestibular approach (1.3%), and open thyroidectomy (0.9%-5.1%)^20–22,29^. We believe that GETTSA provides good identification and observation of the recurrent laryngeal nerves. Injury to the recurrent laryngeal nerve is potentially related to energy conduction and mechanical traction caused by endoscopic equipment. Therefore, an appropriate retraction force and the correct use of ultrasonic knives are particularly important. Owaki et al. reported that the recurrent laryngeal nerve should not come into direct contact when using the blade and recommended that these systems should be used for less than 20 s each time at a distance of 3 mm away from the nerve at level 3^30^. None of the patients in this study experienced permanent vocal cord paralysis. Miscutting of the parathyroid glands is a significant risk factor for hypocalcemia after thyroid surgery. Although endoscopic thyroid surgery has the advantage of a magnification function for identifying the parathyroid glands, preservation of the parathyroid glands, particularly the A2 parathyroid glands, during GETTSA is challenging.^31^ Using carbon nanoparticles may also help to identify and protect the parathyroid glands to some extent^28,31^. Our investigation identified two instances of parathyroid autotransplantation in COT and 10 cases in GETTSA(Table 5). Notably, no symptomatic hypocalcemia occurred in any patient following parathyroid autotransplantation. To better protect the parathyroid glands, we believe that surgeons should strengthen their surgical skill training. Methods such as parathyroid positive imaging and nanocarbon parathyroid negative imaging should be fully utilized. Moreover, the presence or absence of parathyroid glands in specimens subjected to GETTSA must be meticulously ascertained. We recommend using near-infrared autofluorescence imaging of resected specimens to improve the preservation of the parathyroid gland. In cases where inadvertently excised parathyroid glands are identified, reimplantation is recommended to mitigate the risk of postoperative hypoparathyroidism. No postoperative bleeding was observed. Postoperative cough is one of the most important factors leading to bleeding^32^. Therefore, we provided preoperative respiratory management(Including quitting smoking, improving respiratory infections, nebulized inhalation, respiratory training, ect.) and health education to reduce the incidence of postoperative cough. In addition, the rational application of Hemlock clips combined with the use of an ultrasonic scalpel for multisegmental coagulation of vessels also played a role in the intraoperative period. Notably, no patient in our study experienced poor postoperative healing and tracheal fistulas or required secondary surgical intervention. To date, none of the patients included in this study have experienced recurrence, which may be attributed to the relatively mild disease condition of the surgical patients or the short duration of follow-up. Therefore, longer follow-up is necessary to more accurately assess the recurrence rate. Based on the ATA guidelines^23^, we recommend a follow-up period of at least 10 years.

**Table 5:** Postoperative complications.

Variable	COT	GETTSA	X^2^	*P*
Poor incision healing	0	0	-	-
Tracheal fistula	0	0	-	-
Postoperative bleeding	0	0	-	-
Secondary operation	0	0	-	-
Transient vocal cord paralysis			0.503	0.478
Yes	0	2		
No	164	162		
Parathyroid autotransplantation				0.019
Yes	2	10		
No	162	154		

### Limitations

Despite the valuable insights gained from this study, a few limitations must be acknowledged. First, quantifying the total postoperative drainage posed challenges, as the neck drain was not removed when the patient was discharged, resulting in only the first postoperative day’s drainage being recorded. Second, the anatomical location of the parathyroid glands largely influences the difficulty of parathyroid gland protection. However, our analysis did not consider the potential effects of parathyroid gland placement on the procedure. Third, the sample size was insufficient to draw definitive conclusions. Fourth, all surgeons performing procedures in this study had extensive experience with both GETTSA and COT, which likely helped to reduce variability in the results. However, the learning curve can still influence operative time and related complications, and this aspect was not specifically addressed in our study. Finally, this was a retrospective study. Despite using PSM to balance the two groups in terms of the baseline information points, potential selection bias and residual confounding could not be fully ruled out.

## Conclusion

In this study, we compared GETTSA and COT. The results indicated that GETTSA yielded better cosmetic results, swallowing function, and quality of life than COT, but with higher hospitalization costs and longer operative time. When performed with the aid of tracers, GETTSA achieved a level of surgical thoroughness and a rate of postoperative complications comparable to COT. Overall, based on this study, GETTSA is considered a reliable surgical approach. However, due to the relatively short follow-up period and the absence of observed recurrence in this cohort, the potential impact of GETTSA on long-term oncologic control cannot be definitively ascertained. Additionally, due to the lack of sufficient number of patients and multi-center data support, patients undergoing GETTSA must undergo thorough preoperative evaluation to assess the potential for lymph node metastasis and the completeness of dissection. In the future, collecting multicenter data and conducting long-term follow-up will be crucial to more comprehensively evaluate the safety and feasibility of this procedure.

## Data Availability

The data that support the findings of this study are available from the corresponding author upon reasonable request.
